# Narasin Supplementation Enhances Growth Performance in Grazing Cattle

**DOI:** 10.3390/ani15131939

**Published:** 2025-07-01

**Authors:** Daniel M. Polizel, Rodrigo S. Marques, Arnaldo C. Limede, Fernando A. A. Cidrini, José Renato S. Gonçalves, Pedro H. V. Carvalho, Alexandre V. Pires

**Affiliations:** 1 Department of Biology and Animal Science, Sao Paulo State University, Ilha Solteira 15385-000, SP, Brazil; daniel.polizel@unesp.br; 2 School of Animal Sciences, Virginia Polytechnic and State University, Blacksburg, VA 24061, USA; cidrini@vt.edu; 3 Department of Animal Science, College of Agriculture “Luiz de Queiroz”, University of São Paulo, Piracicaba 13418-900, SP, Brazil; fazendafigueira@fealq.com.br (J.R.S.G.); pires.1@usp.br (A.V.P.); 4 Department of Animal Science, Colorado State University, Fort Collins, CO 80523, USA; pedro.carvalho@colostate.edu

**Keywords:** beef cattle, feed additives, forage, grazing, ionophores, mineral supplementation, protein supplementation, forage availability, tropical grass

## Abstract

Three experiments were conducted to evaluate the effects of narasin inclusion on the growth performance of grazing beef cattle under similar forage availability and quality. All three experiments were arranged in a randomized complete block design according to the initial BW. Exp 1: 200 Nellore calves (initial BW = 177 ± 15 kg) were randomly allocated in three treatments for 84 d during the rainy season: (1) CONT: mineral supplement; (2) 1400 N: 1400 mg of narasin/kg of supplement; and (3) 2100 N: 2100 mg of narasin/kg of supplement. Narasin inclusion increased (*p* ≤ 0.01) ADG by 0.077 kg/d and final BW by 6.7 kg, with no differences (*p* ≥ 0.50) between narasin doses. Exp 2: 240 Nellore calves (initial BW = 195 ± 19 kg) were allocated to CONT or N1400 for 112 d during the rainy season. Narasin inclusion increased (*p* ≤ 0.01) ADG by 0.082 kg/d and final BW by 9.1 kg. Exp 3: 150 Nellore yearlings (initial BW = 332 ± 22 kg) were allocated for 112 d during the dry season: (1) PROT: protein supplement and (2) PROT250: inclusion of 250 mg of narasin/kg of supplement. Narasin inclusion increased (*p* ≤ 0.04) ADG by 0.048 kg and final BW by 5.3 kg. For all experiments, narasin inclusion did not impact (*p* ≥ 0.25) supplement intake. Hence, inclusion of narasin in mineral and protein supplements consistently enhances growth performance of grazing beef cattle across different seasons and production stages, without impacting supplement intake, providing an effective nutritional strategy to enhance productivity on tropical pastures.

## Simple Summary

The rearing phase of beef cattle is traditionally carried out in pasture systems in tropical countries. Seasonal variation in the quantity and quality of pasture requires the adoption of nutritional techniques aimed at correcting possible dietary deficiencies to achieve high productivity. The use of supplementation throughout the production cycle is one of the most widely used tools and can be associated with the use of additives. Narasin has been extensively studied in recent years and has shown great potential to enhance beef cattle performance in forage-based diets. The present study evaluated the effects of the inclusion of narasin in mineral supplements and protein supplements on the performance of *Bos indicus* cattle on tropical pasture. Across all three experiments, inclusion of narasin in mineral or protein supplements enhanced average daily gain, consequently resulting in greater final body weight in cattle. Furthermore, the inclusion of narasin did not affect the intake of supplement (mineral or protein), which is of utmost importance to achieve the target daily dose of the additive. In conclusion, narasin supplementation was found to be efficient in increasing grazing beef cattle growth performance during the rainy and dry seasons when added to mineral or protein supplements.

## 1. Introduction

For beef cattle production systems in tropical countries, pastures play an important role, representing the main source of nutrients during the breeding and rearing phases [1]. Pasture management and stocking rate adjustment are essential to optimize animal performance in these locations, where C4 grasses are predominant and have high production potential [2,3,4]. In addition, mineral and protein supplements are adopted strategically to complement nutritional deficiencies, in terms of phosphorus levels, trace minerals, and protein availability during the year [5,6], over and above the possibility of their use as a carrier for feed additives that improve animal performance and health.

Narasin is an ionophore antibiotic naturally produced by the bacteria *Streptomyces aureofaciens*, and studies have shown the efficiency of using this additive on the fermentation and voluntary intake parameters of ruminant animals [7,8,9]. In general, regardless of forage quality, the inclusion of narasin for ruminants fed with forage-based diets increases the molar proportion of ruminal propionate and reduces the ruminal acetate:propionate ratio [10]. Moreover, narasin has shown greater efficacy in modulating the ruminal fermentation in beef cattle fed forage-based diets when compared with other molecular agents, including lasalocid, virginiamycin, salinomycin, and flavomycin [11,12]. Regarding animal growth performance, previous studies have shown that narasin increases dry matter intake (DMI) and average daily gain (ADG) of beef cattle fed forage-based diets [11,12], and offers improvements to the nutritional status of *Bos indicus* heifers during late-gestation [13]. Furthermore, the literature remains limited regarding the evaluation of this molecule’s effects in grazing systems, particularly when administered via supplements. The variability in supplement intake in grazing systems [14,15,16] also introduces a significant challenge in achieving the precise daily dosage required [17,18].

In a study on tropical pastures, including narasin in mineral supplements increased ADG by approximately 200 g during the first 28 d; however, this effect was not maintained over the entire feeding period [14]. In addition, the authors reported a large variation in supplement consumption and pasture availability and quality throughout the study, which may have compromised the potential effect of this feed additive [14]. Therefore, we hypothesized that, under equal conditions of forage availability and quality, including narasin in mineral or protein supplements would increase the performance of grazing beef cattle during a rearing phase. The objective of this study was to evaluate the effects of including narasin in mineral (Exp. 1 and 2; Brazilian rainy season) and protein (Exp. 3; Brazilian dry season) supplements on the performance and supplement intake of grazing beef cattle during the rearing phase.

## 2. Materials and Methods

### 2.1. Ethical Statement

All experimental procedures described in the present study were approved by the Animal Care and Use Committee from the University of São Paulo (CEUA/USP, protocol #5165811516) prior to the initiation of the experiments.

### 2.2. Experimental Location and Overview

The experiments were conducted at the experimental station Agrozootécnica Hildegard Georgina Von Pritzelwiltz, situated in Londrina, Paraná, Brazil (23°34′41″ S, 50°57′08″ W). This facility is owned by the Fundação de Estudos Agrários Luiz de Queiroz (FEALQ, Piracicaba, Brazil), located within a region characterized by a humid subtropical climate (Cfa according to Köppen’s classification), with year-round precipitation [19]. Three experiments were conducted to test the hypotheses of this study.

### 2.3. Experiment 1: Doses of Narasin on Mineral Supplement

Two hundred and forty Nellore calves [initially reduced body weight (BW) = 177 ± 15 kg; age = 8 ± 0.7 mo] were assigned into 30 groups (experimental unit) of eight calves each in a randomized complete block design (10 blocks total), according to their initially reduced BW, which was obtained after 16 h of feed and water withdrawal. Each block contained three groups of eight calves each, with one group randomly assigned to each of the three treatments, resulting in 10 replicates per treatment. The pasture area consisted of 60 paddocks of 1 hectare each, covered with *Urochloa brizantha* cv. Marandu and containing waterers and mineral feeders. Each paddock was continuously grazed by a group for 28 d, followed by a 28 d rest period for that paddock before being reused. The experimental period lasted 84 d, divided into 3 periods of 28 d each during the rainy season (from November 2016 to February 2017).

The groups were randomly assigned to 1 of the 3 following treatments: (1) CONT: mineral supplementation with no fed additives (n = 10); (2) N1400: inclusion of 1400 mg of narasin/kg (Zimprova; Elanco Animal Health, Sao Paulo, Brazil; n = 10) of supplement (dry matter basis) or; (3) N2100: inclusion of 2100 mg of narasin/kg of supplement (dry matter basis; n = 10). The inclusion of narasin in the supplement was carried out to achieve the intake of 13 and 20 ppm of narasin in the total diet (according to manufacturer recommendations), considering the forage intake as being 2.2% of a calf’s BW and the mineral supplement average intake of 40 g/animal daily. Target doses of 13 and 20 ppm were selected based on previous studies demonstrating their effectiveness in modulating ruminal fermentation [20], as well as manufacturer recommendations. The mineral supplement (Phos60; Premix, Ribeirao Preto, SP, Brazil) was used for the CONT, N1400, and N2100 treatments. The composition of the mineral supplement is presented in Table 1.

The supplement and feed refusals were measured and provided once weekly, using a 1.0 g accuracy electronic scale (Toledo 9094C/4; Toledo do Brasil, São Bernardo do Campo, SP, Brazil). Supplements were offered in quantities sufficient to ensure at least 10% refusals, thereby permitting ad libitum intake. Samples of the supplement offered and the refusals were collected to determine the DM content [21] for the calculation of the average DM supplement intake.

The animals were weighed at the beginning of the experiment (d 0) and at the end of each period (d 28, 56, and 84) after 16 h of feed and water withdrawal using the idBeck 3.0 electronic scale (Irmãos Beckhauser e Cia Ltda, Paranavaí, PR, Brazil). The ADG (kg/d) was calculated by dividing the gain obtained by the duration of each period (28 d).

Total forage availability in the paddocks was assessed at the entry and exit of the animals on d 1 and 28 of each period, respectively. The quantitative samples were harvested near the ground using 0.25 m^2^ metallic frames (0.5 × 0.5 m) placed on the representative sites. The samples obtained were sent to the laboratory for subsequent determination of the DM content and calculation of forage availability per hectare. On d 14 of each experimental period, forage samples were collected from each paddock using simulated grazing to assess forage quality.

The forage samples were dried in a forced-air oven at 55 °C for 96 h and subsequently ground in a 1 mm screen using a Willey mill (Marconi Equipaments Laboratories, Piracicaba, SP, Brazil). The DM was determined by drying the samples at 105 °C in an oven for 24 h [22], and the ash content was determined by incinerating the samples in a muffle furnace at 550 °C [22]. The organic matter (OM) was calculated using the following equation: OM = 100 − ash. Total nitrogen was determined using a LECO TruMac N (Leco Corporation; Saint Joseph, MI, USA; [22]), and the crude protein (CP) was obtained by multiplying the total N content by 6.25. The neutral detergent fiber (NDF; [23]) and acid detergent fiber (ADF; [24]) were determined using an Ankom 2000 fiber analyzer (Ankom Tech. Corp., Macedon, NY, USA). Sodium sulfite and heat-stable α-amylase were added in the NDF analysis.

All data were analyzed for normality of residuals using the Shapiro–Wilk test, homogeneity of variances using the Levene test, and removal of outliers based on the student’s r value. The data were analyzed using Kenward–Roger approximation to determine the denominator df for the test of fixed effects. The block was considered as a random effect. The covariance structure adopted was first-order autoregressive, which provided the smallest Akaike information criterion corrected (AICC) for the variables analyzed. To evaluate the effect of the treatments, 2 orthogonal contrasts were previously proposed: (1) CONT vs. N: supplement with no feed additives versus supplements with narasin (1400 and 2100 mg/kg of supplement) and (2) N1400 vs. N2100: comparison between doses of 1400 and 2100 mg/kg of supplement. Significance was set at *p* ≤ 0.05, and tendencies were determined if *p* > 0.05 and ≤0.10. Results are reported according to the main effects if no interactions were significant.

### 2.4. Experiment 2: Narasin on Mineral Supplement

Two hundred and forty weaned Nellore calves [initially reduced BW = 195 ± 19 kg; age = 8 ± 0.5 mo] were assigned into 8 groups (experimental unit) of six calves each and 12 groups of eight calves each in a randomized complete block design (10 blocks total), according to their initially reduced BW, obtained after 16 h of feed and water withdrawal. Each block contained two groups of either six or eight calves each, with one group randomly assigned to each of the two treatments, resulting in 10 replicates per treatment. The pasture area consisted of 62 paddocks of 1 hectare each, covered with *Urochloa brizantha* cv. Marandu and containing waterers and mineral feeders. Each paddock was continuously grazed by a group for 28 d, followed by a 28 d rest period for that paddock before being reused. The experimental period lasted 112 d, divided into 4 periods of 28 d each during the wet season (from November 2017 to March 2018). The rotation of the groups in the paddocks was carried out to ensure that the animals from both treatments passed through the same paddocks, minimizing the location effect.

The groups were randomly assigned to 1 of the 2 following treatments: (1) CONT: mineral supplementation with no feed additives or (2) N1400: inclusion of 1400 mg of narasin/kg of mineral supplement. The inclusion of narasin in the supplement was carried out to achieve the intake of 13 ppm of narasin in the total diet, considering the forage intake as being 2.2% of BW and the mineral supplement intake of 40 g/animal daily. The mineral supplement (Phos60; Premix, Ribeirao Preto, SP, Brazil) was used for the CONT and N1400 treatments. The composition of the supplement is presented in Table 1.

The animals were weighed at the beginning of the experiment (d 0) and at the end of each period (d 28, 56, 84, and 112) after a 16 h feed and water withdrawal, using the idBeck 3.0 electronic scale (Irmãos Beckhauser e Cia Ltda, Paranavaí, PR, Brazil). The ADG was calculated by dividing the total gain obtained by the duration of each period (28 d). The assessments of pasture availability and quality, as well as the chemical analyses performed, were carried out in the same manner as described in Exp. 01.

All data were analyzed for normality of residuals using the Shapiro–Wilk test, homogeneity of variances using the Levene test, and removal of outliers based on the student’s r value. The data were analyzed using Kenward–Roger approximation to determine the denominator df for the test of fixed effects. The block was considered as a random effect. The covariance structure adopted was first-order autoregressive, which provided the smallest Akaike information criterion corrected (AICC) for the variables analyzed. The treatment effect was defined by the F test. Significance was set at *p* ≤ 0.05, and tendencies were determined if *p* > 0.05 and ≤0.10. Results are reported according to the main effects if no interactions were significant.

### 2.5. Experiment 3: Narasin on Protein Supplement

One hundred and fifty weaned Nellore yearlings [initially reduced BW = 332 ± 22 kg; age 16 ± 0.9 mo] were assigned into 30 groups of five yearlings each in a randomized complete block design (15 blocks total), according to their reduced BW, obtained after 16 h of feed and water withdrawal. Each block contained two groups of five yearlings each, with one group randomly assigned to each of the two treatments, resulting in 15 replicates per treatment. The pasture area consisted of 62 paddocks of 1 hectare each, covered with *Urochloa brizantha* cv. Marandu and containing waterers and feeders. Pasture management and group rotation between paddocks were the same as described in Exp. 02. The experimental period lasted 112 d, divided into 4 periods of 28 d each during the dry season (from May 2018 to August 2018).

The groups were randomly assigned to one of the two following treatments: (1) PROT: protein supplementation with no feed additives, or (2) PROT250: inclusion of 250 mg of narasin/kg of protein supplement. The inclusion of narasin in the supplement was carried out with the objective of achieving an intake of 13 ppm of narasin in the total diet, considering the forage intake as being 2.2% of BW and protein supplement intake of 450 g/animal daily. The protein supplement (Campo 30; Premix, Ribeirao Preto, SP, Brazil) was used for the PROT and PROT250 treatments. The composition of the supplement is presented in Table 1.

The assessments of pasture availability and quality, as well as the chemical analyses performed, were carried out in the same manner as described in Exp. 01. Statistical analysis of the data was performed as described in Exp. 02.

## 3. Results

### 3.1. Exp. 1

**Figure 1:**
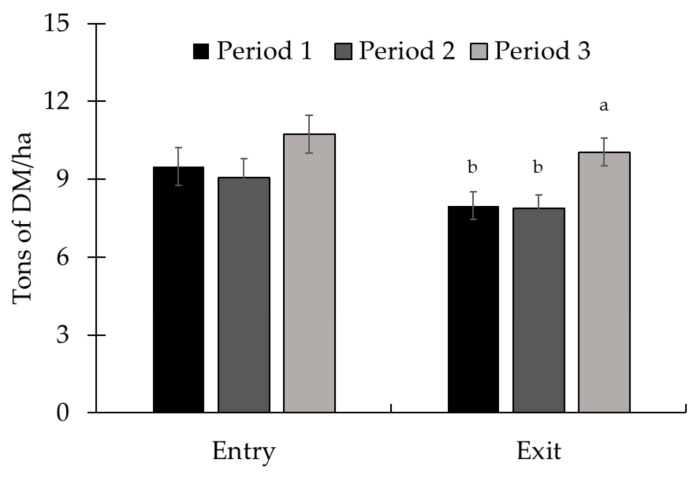
Forage mass availability at the entrance (d 0) and exit of animals (d 28) during experiment 1. There was no period effect for the availability at the entrance (*p* = 0.11; Table 1); however, the availability of mass at the exit was greater in period 3 in relation to the other periods (*p* < 0.01). Total forage availability in the paddocks was assessed at the entry and exit of the animals, i.e., on d 1 and 28 of each period, respectively. The quantitative samples were harvested close to the ground using 0.25 m^2^ metallic frames (0.5 × 0.5 m) placed on the representative sites. The samples obtained were sent to the laboratory for subsequent determination of the dry matter content and calculation of forage availability per hectare. Means without a common superscript letter differ (*p* < 0.05).

**Figure 2:**
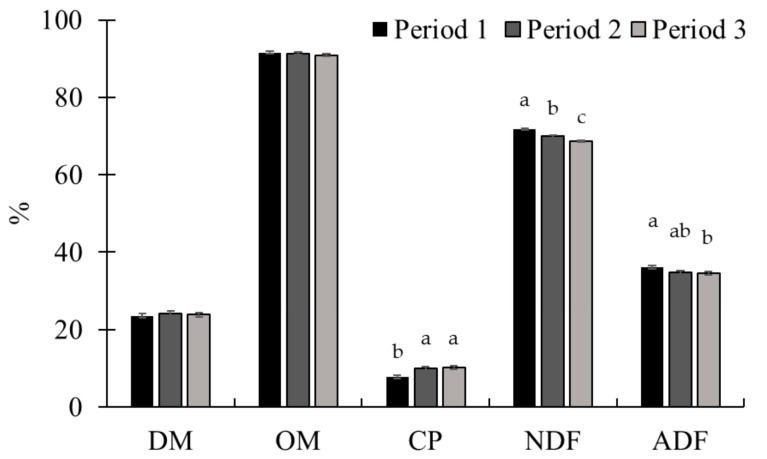
Chemical composition of pasture throughout experiment 1. There was no effect of period for dry matter (DM) and organic matter (OM) content (*p* ≥ 0.39). Crude protein (CP) content was higher in periods 2 and 3 (*p* < 0.01). Neutral detergent fiber (NDF) and acid detergent fiber (ADF) content decreased throughout the experimental periods (*p* < 0.01). To assess the quality of the forage available in the paddocks, on d 14 of each experimental period, a sample was obtained per paddock through simulated grazing. The DM was determined by drying the samples at 105 °C in an oven for 24 h (AOAC, 1997 [22]), and the ash content was determined by burning the samples in a muffle furnace at 550 °C (AOAC, 1997 [22]). The OM was calculated using the following equation: OM = 100 − ash. Total nitrogen determination was performed using a LECO TruMac N (Leco Corporation; Saint Joseph, MI, USA; AOAC, 1997 [22]) and the CP was obtained by multiplying the total N content by 6.25. The NDF (Van Soest et al., 1991 [23]) and ADF (Goering and Van Soest, 1970 [24]) were determined using an Ankom 2000 fiber analyzer (Ankom Tech. Corp., Macedon, NY, USA). Sodium sulfite and heat-stable α-amylase were added in the NDF analysis. Within nutrients, means without a common superscript letter differ (*p* < 0.05).

There was no interaction (*p* ≥ 0.15) between treatment and period for forage availability and chemical composition of the paddocks in this study (Table 2). Forage availability and quality at the beginning and end of the periods were similar (*p* ≥ 0.45) among treatments. Forage availability at the animals’ exit was greater (*p* < 0.01) in period 3 compared with the other periods. There was a period effect (*p* ≤ 0.03) on forage availability at exit (Figure 1) and forage CP, NDF, and ADF content (Figure 2). There was no effect of period for DM and OM content (*p* ≥ 0.39). The CP content was higher in periods 2 and 3 (*p* < 0.01) compared with period 1. The NDF and ADF content decreased throughout the experimental periods (*p* < 0.01).

The inclusion of narasin (N1400 or N2100) in the mineral supplement increased (*p* < 0.01) ADG compared with CONT, with no difference between narasin supplementation treatments (Table 3). Consequently, the inclusion of N1400 or N2100 tended (*p* = 0.06) to increase the BW of the animals after 28 d of study and increased (*p* < 0.01) BW on d 56 and 84 compared with CONT (Table 3). The doses of narasin increased the BW changes during the study (*p* < 0.01) compared with CONT, with no differences (*p* = 0.50) between N1400 and N2100 (Table 3). The inclusion of narasin in the mineral supplement did not affect (*p* ≥ 0.18) supplement intake (Table 3).

**Figure 3:**
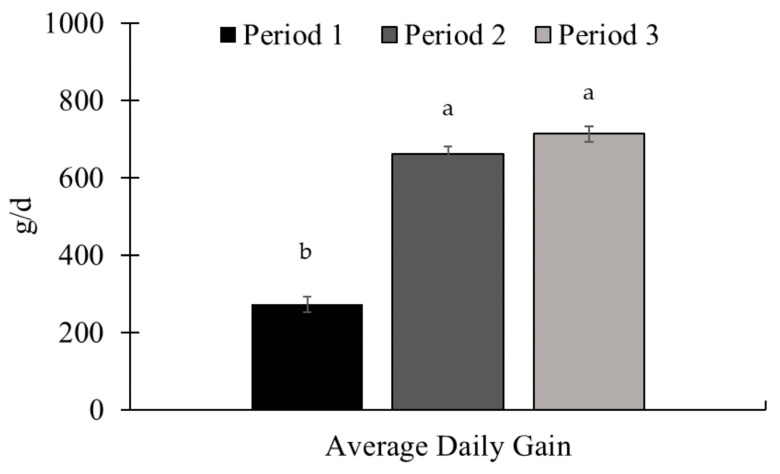
Average daily gain during the experimental periods (Exp. 01). There was a period effect for average daily gain (ADG), with the largest gains observed in periods 2 and 3 (*p* < 0.01). The animals were weighed at the beginning of the experiment (d 0) and at the end of each period (d 28, 56, and 84) after a 16 h feed and water withdrawal, using the idBeck 3.0 electronic scale (Irmãos Beckhauser e Cia Ltda, Paranavaí, PR, Brazil). The ADG (kg/d) was calculated by dividing the gain obtained by the duration of each period (28 d). Means without a common superscript letter differ (*p* < 0.05).

**Figure 4:**
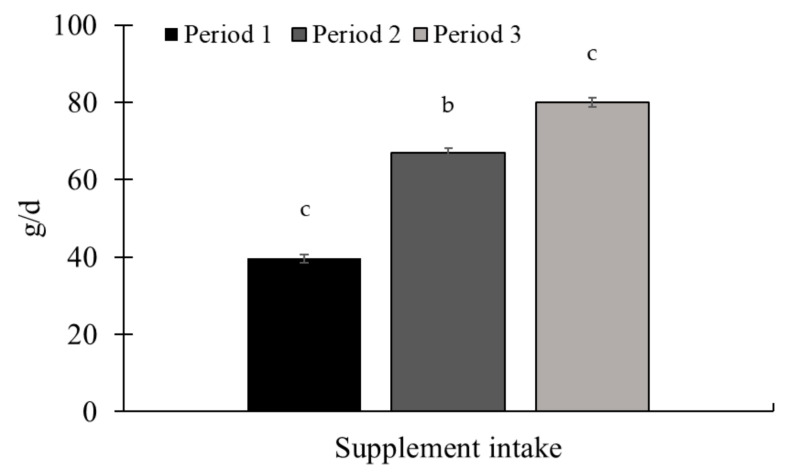
Mineral supplement intake throughout Exp. 1. There was an increase in supplement intake between the experimental periods (*p* < 0.01). The supplement was provided once a week, as well as the measurement of the refusal. The amount of supplement offered, and the refusal were quantified on the Toledo 9094C/4 electronic scale, accurate to 1.0 g (Toledo do Brasil, São Bernardo do Campo, SP, Brazil). Supplements were offered in quantities sufficient to ensure at least 10% refusals, thereby permitting ad libitum intake. Samples of the supplement offered, and the leftovers were collected to determine the dry matter content (AOAC, 2000 [21]) for later calculation of the average supplement intake in the dry matter. Means without a common superscript letter differ (*p* < 0.05).

There was an effect of the experimental period for ADG (*p* < 0.01; Figure 3) and supplement intake (*p* < 0.01; Figure 4), with the highest ADG being observed in periods 2 and 3. The supplement intake increased throughout the experiment, with the highest intake observed in period 3.

### 3.2. Exp. 2

**Figure 5:**
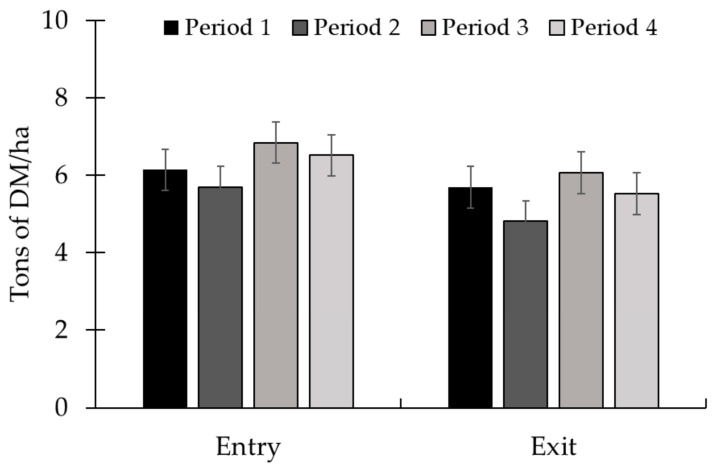
Availability of forage mass at the entrance (d 0) and exit of the animals of the paddock (d 28) throughout Exp. 2. There was no period effect (*p* ≥ 0.10) on forage mass availability. Total forage availability in the paddocks was assessed at the entry and exit of the animals, on d 1 and 28 of each period, respectively. The quantitative samples were harvested close to the ground using 0.25 m^2^ metallic frames (0.5 × 0.5 m) placed on the representative sites. The samples obtained were sent to the laboratory for subsequent determination of the dry matter content and calculation of forage availability per hectare.

**Figure 6:**
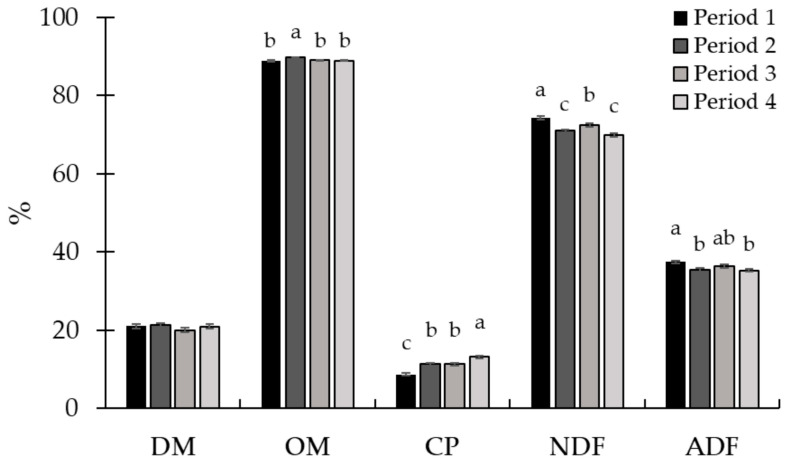
Chemical composition of pasture in experimental periods (Exp. 2). There was no period effect on dry matter (DM) content. The highest organic matter (OM) content was observed in period 2 (*p* < 0.01). The crude protein (CP) content increased throughout the experiment (*p* < 0.01). The highest neutral detergent fiber (NDF) and acid detergent fiber (ADF) contents were observed in period 1, and the lowest were observed in periods 2 and 4 (*p* < 0.01). To assess the quality of the forage available in the paddocks, on d 14 of each experimental period, a sample was obtained per paddock through simulated grazing. The DM was determined by drying the samples at 105 °C in an oven for 24 h (AOAC, 1997 [22]), and the ash content was determined by burning the samples in a muffle furnace at 550 °C (AOAC, 1997 [22]). The OM was calculated using the following equation: OM = 100 − ash. Total nitrogen determination was performed using a LECO TruMac N (Leco Corporation; Saint Joseph, MI, USA; AOAC, 1997 [22]), and the CP was obtained by multiplying the total N content by 6.25. The NDF (Van Soest et al., 1991 [23]) and ADF (Goering and Van Soest, 1970 [24]) were determined using an Ankom 2000 fiber analyzer (Ankom Tech. Corp., Macedon, NY, USA). Sodium sulfite and heat-stable α-amylase were added in the NDF analysis. Within nutrients, means without a common superscript letter differ (*p* < 0.05).

No treatment × period interaction (*p* ≥ 0.16; Table 4) was observed for pasture and animal performance data. The availability and chemical composition of the grass were the same between treatments (*p* ≥ 0.18). There was no period effect (*p* ≥ 0.10) on forage mass availability and DM content (Figure 5). The greatest forage OM content was observed in period 2, with no difference between the other periods. The least forage CP content occurred in period 1, while the greatest CP forage value was observed in period 3. The NDF and ADF contents were greater in the first period and smaller in periods 2 and 4 (Figure 6).

The inclusion of 1400 mg of narasin per kg of mineral supplement increased (*p* < 0.01) ADG during the experiment (Table 5). Thus, the BW of calves fed N1400 tended (*p* = 0.08) to increase on d 28 of the study and increased (*p* < 0.01) on d 56, 84, and 112 compared with CONT (Table 5). Consequently, the BW change was greater (*p* < 0.01) for the N1400 compared with CONT (Table 5). The inclusion of narasin did not affect (*p* = 0.25) the daily intake of mineral supplements (Table 5).

**Figure 7:**
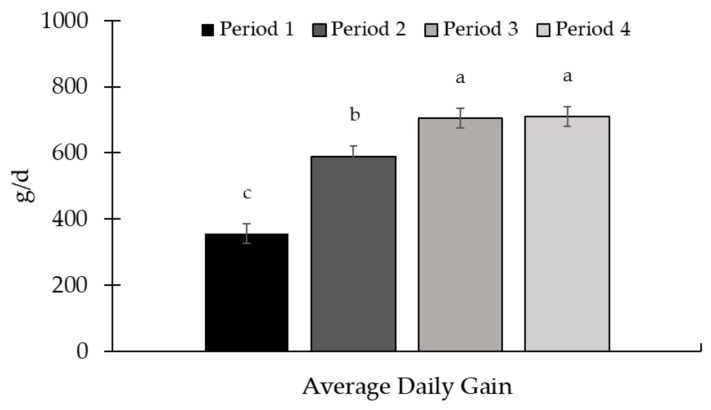
Average daily gain in the experimental periods (Exp. 2). There was a period effect (*p* < 0.01) with the highest gains observed in periods 3 and 4, and the lowest ADG observed in period 1. The animals were weighed at the beginning of the experiment (d 0) and at the end of each period (d 28, 56, 84, and 112) after a 16h feed and water withdrawal, using the idBeck 3.0 electronic scale (Irmãos Beckhauser e Cia Ltd.a, Paranavaí, PR, Brazil). The ADG (kg/d) was calculated by dividing the gain obtained by the duration of each period (28 d). Means without a common superscript letter differ (*p* < 0.05).

**Figure 8:**
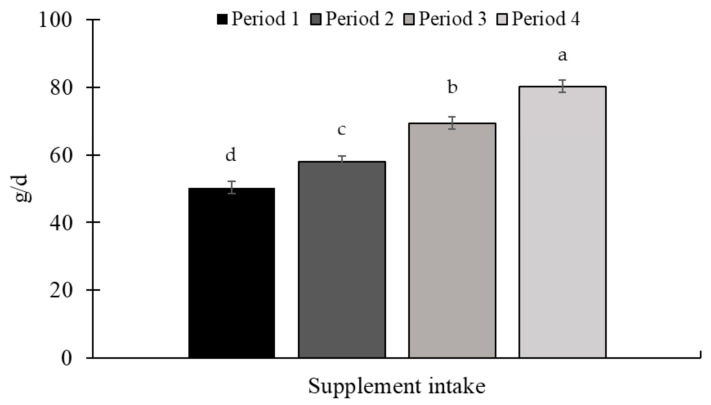
Mineral supplement intake throughout Exp. 2. There was a period effect (*p* < 0.01) with an increase in supplement intake during the study. The supplement was provided once a week, as well as the measurement of the refusal. The amount of supplement offered, and the refusal were quantified on a Toledo 9094C/4 electronic scale accurate to 1.0 g (Toledo do Brasil, São Bernardo do Campo, SP, Brazil). Supplements were offered in quantities sufficient to ensure at least 10% refusals, thereby permitting ad libitum intake. Samples of the supplement offered, and the leftovers were collected to determine the dry matter content (AOAC, 2000 [21]) for later calculation of the average supplement intake in the dry matter. Means without a common superscript letter differ (*p* < 0.05).

A period effect (*p* < 0.01) was observed for ADG and supplement intake (Figure 7 and Figure 8, respectively). The highest ADG was observed in periods 3 and 4, while the lowest gain was observed in period 1. Supplement intake increased throughout the experiment.

### 3.3. Exp. 3

**Figure 9:**
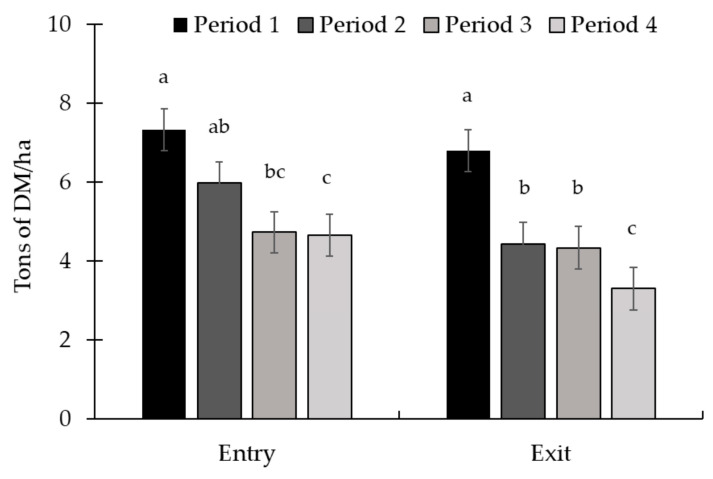
Forage mass availability at the entry (d 0) and exit (d 28) periods in Exp. 3. Forage mass availability decreased throughout the experiment at the entry (*p* < 0.01) and exit (*p* < 0.01). Total forage availability in the paddocks was assessed at the entry and exit of the animals, i.e., on d 1 and 28 of each period, respectively. The quantitative samples were harvested close to the ground using 0.25 m^2^ metallic frames (0.5 × 0.5 m) placed on the representative sites. The samples obtained were sent to the laboratory for subsequent determination of the dry matter content and calculation of forage availability per hectare. Means without a common superscript letter differ (*p* < 0.05).

**Figure 10:**
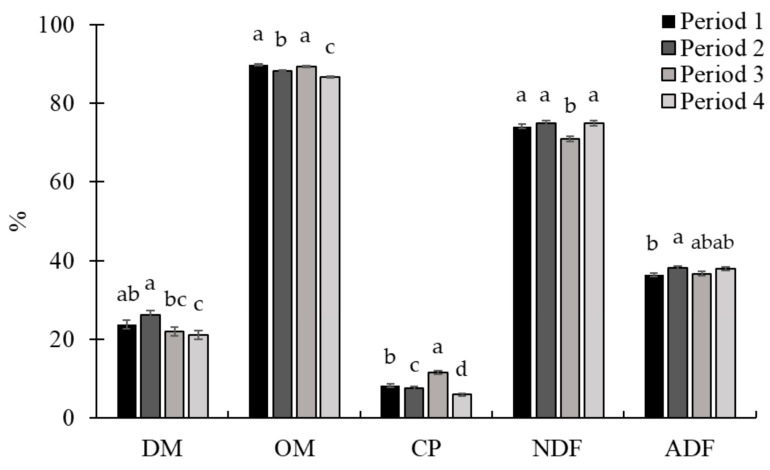
Chemical composition of pasture throughout Exp. 3. There was a period effect (*p* ≤ 0.03) for all variables evaluated. The highest dry matter (DM) content was observed in period 2, while the lowest value occurred in period 4. The highest organic matter (OM) values were observed in periods 1 and 3. Regarding crude protein (CP), the highest value occurred in period 3 and the lowest value in period 4. The highest neutral detergent fiber (NDF) contents occurred in periods 1, 2, and 4. The highest acid detergent fiber (ADF) content occurred in period 2 and the lowest in period 3, with intermediate values in periods 3 and 4. To assess the quality of the forage available in the paddocks, on d 14 of each experimental period, a sample was obtained per paddock through simulated grazing. The DM was determined by drying the samples at 105 °C in an oven for 24 h (AOAC, 1997 [22]), and the ash content was determined by burning the samples in a muffle furnace at 550 °C (AOAC, 1997 [22]). The OM was calculated using the following equation: OM = 100 − ash. Total nitrogen determination was performed using a LECO TruMac N (Leco Corporation; Saint Joseph, MI, USA; AOAC, 1997 [22]) and the CP was obtained by multiplying the total N content by 6.25. The NDF (Van Soest et al., 1991 [23]) and ADF (Goering and Van Soest, 1970 [24]) were determined using an Ankom 2000 fiber analyzer (Ankom Tech. Corp., Macedon, NY, USA). Sodium sulfite and heat-stable α-amylase were added in the NDF analysis. Within nutrients, means without a common superscript letter differ (*p* < 0.05).

No treatment × period interaction (*p* ≥ 0.38; Table 6) was observed for pasture and animal performance. The availability of forage at the entrance and exit of the animals from the paddock, as well as the grass quality, were similar between treatments (*p* ≥ 0.58). There was a period effect (*p* ≤ 0.03) for all variables related to forage mass and composition. Forage mass availability at entry and exit decreased (*p* < 0.01) throughout the experiment (Figure 9). The greatest forage DM content occurred in period 2, while the lowest value was in period 4 (Figure 10). Forage OM content was greater in periods 1 and 3, and the lowest value was in period 4. Pasture CP was highest in period 3, while the lowest value was observed in period 4. The lowest NDF content was observed in period 3, with no difference between the other periods. Additionally, in relation to ADF, the highest value occurred in period 2 and the lowest value in period 1.

The ADG increased (*p* < 0.01; Table 7) with the inclusion of narasin in the protein supplement. After 28 d of supplementation, BW was similar (*p* = 0.29) between treatments. On d 56, however, BW tended to be greater (*p* = 0.08) in PROT250 vs. PROT (Table 7). From d 84 to 112, PROT250 yearlings were heavier (*p* ≤ 0.02) compared with yearlings from the PROT group. The addition of narasin to the protein supplement did not affect (*p* = 0.74) supplement intake (Table 7).

**Figure 11:**
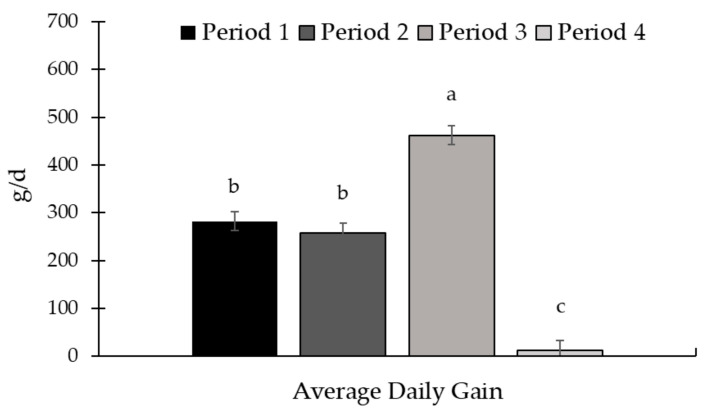
Average daily gain in the experimental periods (Exp. 03). There was a period effect for average daily gain (ADG; *p* < 0.01), with the highest gain in period 3 and the lowest gain in period 4. The animals were weighed at the beginning of the experiment (d 0) and at the end of each period (d 28, 56, 84, and 112) after a 16 h feed and water withdrawal, using the idBeck 3.0 electronic scale (Irmãos Beckhauser e Cia Ltd.a, Paranavaí, PR, Brazil). The ADG (kg/d) was calculated by dividing the gain obtained by the duration of each period (28 d). Means without a common superscript letter differ (*p* < 0.05).

**Figure 12:**
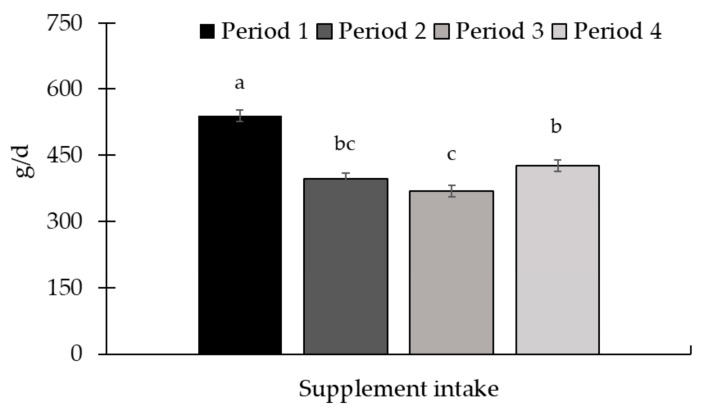
Protein supplement intake throughout Experiment 3. There was a period effect (*p* < 0.01) for supplement intake, with higher intake in period 1 and lower in period 3. The supplement was provided once a week, as well as the measurement of the refusal. The amount of supplement offered, and the refusal, were quantified on a Toledo 9094C/4 electronic scale, accurate to 1.0 g (Toledo do Brasil, São Bernardo do Campo, SP, Brazil). Supplements were offered in quantities sufficient to ensure at least 10% refusals, thereby permitting ad libitum intake. Samples of the supplement offered, and the leftovers, were collected to determine the dry matter content (AOAC, 2000 [21]) for later calculation of the average supplement intake in the dry matter. Means without a common superscript letter differ (*p* < 0.05).

There was a period effect for ADG (*p* < 0.01; Figure 11) and supplement intake (*p* < 0.01; Figure 12). The greatest ADG was observed for period 3, while the lowest ADG occurred in the last period of the study. Regarding supplement intake, the highest values were observed in period 1, while the lowest were in period 3.

## 4. Discussion

The benefits of including ionophores in ruminant diets are well documented in the literature, demonstrating positive effects on ruminal fermentation [25,26], performance [27,28,29,30], and coccidiosis control [31,32]. It is important to highlight that there is a greater number of scientific investigations on the use of these molecules in diets containing a high concentrate content (feedlot cattle), with a less significant number of studies examining grazing cattle. In addition, beef producers need to be aware of the particularities of each ionophore to decide which one to use in their production system [7,33,34]. Therefore, the present study established an important database on the inclusion of narasin in two types of supplementations (mineral and protein) for animals in a tropical pasture system and at different seasons of production (dry and rainy).

Several studies have demonstrated the positive results of narasin on the performance of ruminants in different production systems, such as increased milk production in sheep [35], increased ADG and FE of feedlot lambs [36], and in the rearing of cattle fed forage-based diets [11,12]. Additionally, narasin is an ionophore that modulates rumen fermentation dynamics [11], alters plasma metabolite profiles by increasing glucose concentrations [35] and reducing urea levels, and improves animal performance [20]. In the current study, the inclusion of narasin in the supplement increased ADG in the three experiments conducted in a grazing system. The observed improvements in cattle growth performance can be explained by the ability of narasin to modulate ruminal fermentation, increasing the energy efficiency due to the higher molar proportion of propionate and the reduction in the acetate:propionate ratio [20]. Furthermore, studies conducted with feedlot cattle fed forage-based diets [11,12] have shown that narasin supplementation increases DMI, thereby enhancing total organic matter intake. This increase contributes to greater ruminal fermentation and, consequently, a higher energy supply to support animal performance [11,12]. Therefore, this combination of fermentation manipulation, and a possible increase in DMI and plasma metabolites (glucose) could explain the increase in animal weight gain, as all treatments were in pasture systems with the same forage availability and quality.

Providing low-intake supplements is a simple and low-cost way to provide additives to animals raised in pasture-based systems [37,38]. However, in order to achieve the recommended intake dose of the additive, supplement intake cannot be affected (positively or negatively) by the inclusion of the additive, as this could result in over- or under-dosage. The ADG increases quadratically with increasing doses of monensin or lasalocid, emphasizing the importance of consuming the correct dose to optimize animal performance [37]. Some additives appear to affect the supplement intake, varying the additive intake. Evaluation of additive inclusion in mineral supplements for cattle on tropical pasture revealed that supplementation with 1000 mg monensin/kg or 1111 mg salinomycin/kg reduced supplement intake by 38.4% and 27.5%, respectively, compared with the control [39]. Franco [40] reported that the inclusion of monensin in mineral supplements not only reduced supplement intake but also decreased the frequency of animal visits to the trough. Additionally, aversion to monensin-containing supplements increased over time, suggesting a conditioned response to post-ingestive effects [40]. The first study to evaluate the use of narasin in mineral supplementation was carried out by our research group [41], in which the inclusion of 650 or 1300 mg of narasin/kg of mineral supplement did not affect supplement intake, and the higher dose increased the ADG of feedlot heifers fed high forage content diets. In the three experiments reported in this study, the inclusion of narasin in mineral and protein supplements did not alter supplement intake, thus allowing the expected dose of narasin to be consumed during the study.

It is important to emphasize that the absence of interaction between treatment and experimental period in all experiments carried out in this study demonstrates that, based on only on the quantitative and qualitative variations measured and presented in the figures of this manuscript, the use of narasin was effective in ensuring an increase in animal performance, with no effect on supplement intake. Limede et al. [11] reported variations in the composition of forage provided to feedlot cattle supplemented with 13 ppm of narasin and also observed that, despite this variation, the effect of narasin on performance was maintained, providing additional gains in relation to the control treatment of approximately 110 g/d. Studies have demonstrated that narasin is capable of positively manipulating the ruminal fermentation, with an increase in propionate and a reduction in the acetate:propionate ratio even with variations in the nutritional quality of forages for both sheep [36] and cattle [10], which may explain the results in animal performance obtained in the present study.

**Figure 13:**
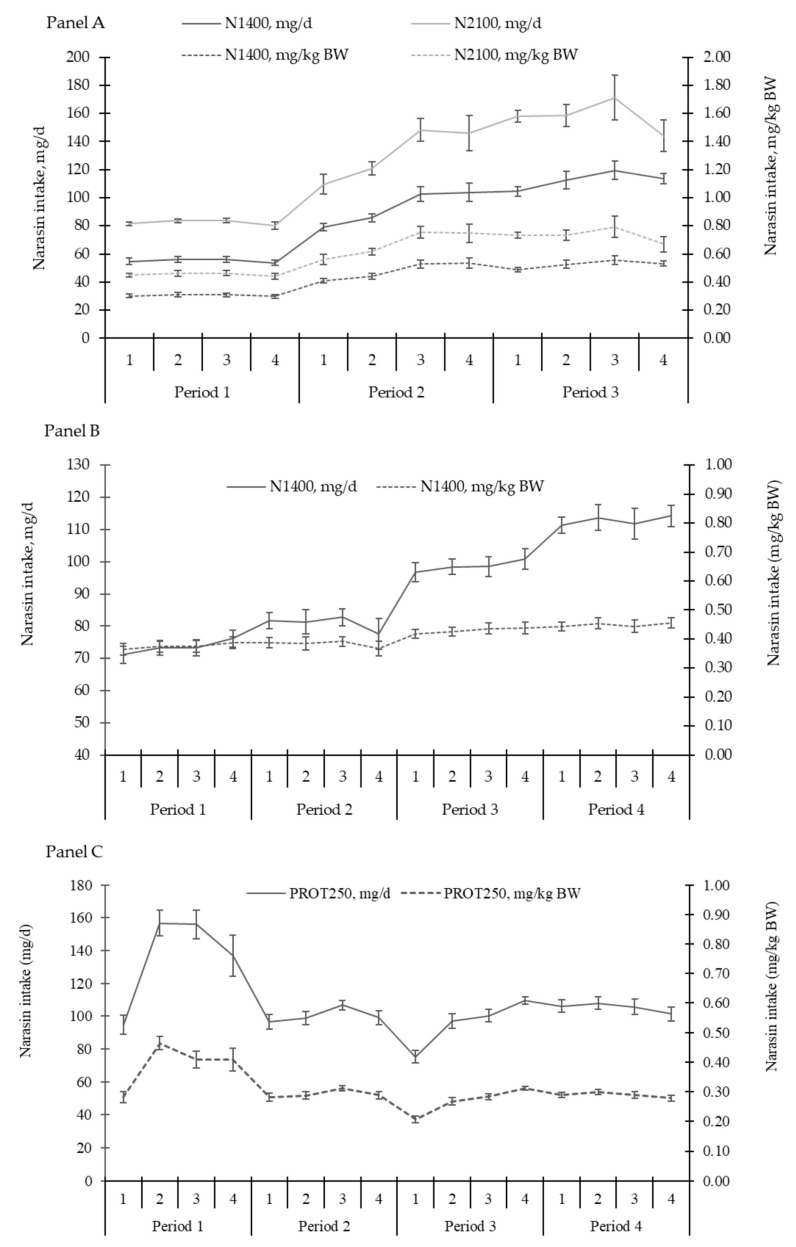
Panel (**A**)—Narasin intake over the experimental periods of Exp. 1, expressed in mg/d and mg/kg of body weight, calculated from the supplement consumption and narasin concentration. Panel (**B**)—Narasin intake over the experimental periods of Exp. 2, expressed in mg/d and mg/kg of body weight, calculated from the supplement consumption. Panel (**C**)—Narasin intake over the experimental periods of Exp. 3, expressed in mg/d and mg/kg of body weight, calculated from the supplement consumption.

Throughout the three experiments, variation in supplement consumption was also observed. Many factors can affect supplement consumption, such as soil fertility, season, forage quality, protein and energy availability, water quality, and palatability of the mineral supplement [38]. The variation in consumption may be influenced by the type of supplement, as presented by [42], reporting that 25.8% of the animals visited the feeder daily when fed the mineral supplement; however, when a protein-energetic supplement was offered, the visit increased to 85.1%. The issue to be highlighted is that supplements were the vehicles for supplying narasin, therefore, variations in supplement intake result in variations in additive intake, as shown in Figure 13. Despite this, narasin showed a consistent effect on animal performance in all experiments (absence of treatment × period interaction). Previous studies have shown that narasin is capable of manipulating ruminal fermentation even if the frequency of intake of the molecule is reduced, that is, increasing the interval between supplying the molecule [43]. Furthermore, it is possible to observe residual effects of the manipulation on ruminal fermentation up to 3 d after the withdrawal of narasin from the diet of *Bos indicus* cattle fed with a high forage content [43].

## 5. Conclusions

In summary, this study demonstrated that, under similar forage quality and availability, narasin supplementation improves animal performance without altering the intake of protein or mineral supplements. These findings support the use of narasin as a practical strategy to enhance productivity in beef cattle grazing systems, with potential application across both tropical rainy and dry seasons.

## Figures and Tables

**Table 1:** Nutritional and mineral composition of the supplements used during experiments 1, 2, and 3 ^1,2^.

Item	Experiments 1 and 2			Experiment 3	
	CONT	N1400	N2100	PROT	PROT250
g.kg^−1^					
Calcium	120.0	120.0	120.0	60.0	60.0
Phosphorus	60.0	60.0	60.0	15.0	15.0
Sodium	145	145	145	72.0	72.0
Sulfur	10.0	10.0	10.0	7.0	7.0
mg.kg^−1^					
Copper	1200	1200	1200	228.8	228.8
Zinc	3450	3450	3450	800.0	800.0
Iodine	80.0	80.0	80.0	28.0	28.0
Cobalt	80.0	80.0	80.0	42.0	42.0
Selenium	20.0	20.0	20.0	5.0	5.0
Narasin	0	1400	2100	0	250
Crude protein, % of DM	-	-	-	30.0	30.0

^1^ Exp 1: Two hundred and forty Nellore calves were assigned into 30 groups of eight calves each and randomly assigned to one of the three following treatments: (1) mineral supplementation with no fed additives [CONT, n = 10]; (2) inclusion of 1400 mg of narasin/kg of supplement [N1400, n = 10] and; (3) inclusion of 2100 mg of narasin/kg of supplement [N2100, n = 10]. The experimental period lasted 84 d, divided into 3 periods of 28 d each during the rainy season (from November 2016 to February 2017). Exp 2: Two hundred and forty weaned Nellore calves were assigned into 8 groups of six calves each and 12 groups of eight calves each and randomly assigned to one of the two following treatments: (1) mineral supplementation with no fed additives [CONT, n = 10] or (2) inclusion of 1400 mg of narasin/kg of supplement [N1400, n = 10]. The experimental period lasted 112 d, divided into 4 periods of 28 d each during the rainy season (from November 2017 to March 2018). ^2^ One hundred and fifty weaned Nellore yearlings were assigned into 30 groups of five animals each and randomly assigned to one of the two following treatments: (1) protein supplementation with no feed additives [PROT, n = 15] or (2) protein supplementation with inclusion of 250 mg of narasin/kg of protein supplement [PROT250, n = 15]. The experimental period lasted 112 d, divided into 4 periods of 28 d each during the dry season (from May 2018 to August 2018).

**Table 2:** Forage availability and chemical composition of the paddocks covered by *Urochloa brizantha* cv. Marandu in Exp. 1.

Item	Treatments ^1^			SEM	*p*-Value				
	CONT	N1400	N2100		TRT	CONT vs. NAR	N1400 vs. N2100	Period	T × P
Forage availability, T DM/ha ^2^									
Initial	9.95	9.38	9.96	0.61	0.65	0.65	0.42	0.11	0.15
Final	8.67	8.27	8.97	0.41	0.45	0.91	0.21	<0.01	0.22
Chemical composition, % ^3^									
Dry matter	24.16	23.64	23.79	0.41	0.56	0.30	0.76	0.59	0.23
Organic matter	91.25	91.37	91.09	0.29	0.71	0.94	0.41	0.39	0.46
Crude protein	9.33	9.32	9.29	0.27	0.91	0.77	0.76	<0.01	0.19
Neutral detergent fiber	70.23	70.15	70.13	0.19	0.86	0.59	0.94	<0.01	0.73
Acid detergent fiber	35.20	35.36	35.11	0.35	0.78	0.91	0.49	0.03	0.83

^1^ CON = mineral supplementation without feed additives; N1400 = inclusion of 1400 mg of narasin/kg of supplement; N2100 = inclusion of 2100 mg of narasin/kg of supplement. ^2^ Total forage availability in the paddocks was assessed at the entry and exit of the animals, i.e., on d 1 and 28 of each period, respectively. The quantitative samples were harvested close to the ground using 0.25 m^2^ metallic frames (0.5 × 0.5 m) placed on the representative sites. ^3^ The samples obtained were sent to the laboratory for subsequent determination of the dry matter content and calculation of forage availability per hectare.

**Table 3:** Experiment 1: Performance and supplement intake of Nellore calves receiving a mineral supplementation with no feed additives (CONT) or inclusion of 1400 (N1400) or 2100 (N2100) mg of narasin/kg of supplement.

Item	Treatments ^1^			SEM	*p*-Value				
	CONT	N1400	N2100		TRT	CONT vs. NAR	N1400 vs. N2100	Period	T × P
Body weight, kg ^2^									
Initial	176.9	176.9	176.6	0.20	0.57	0.58	0.37	-	-
28 d	182.6	184.7	186.0	1.14	0.12	0.06	0.41	-	-
56 d	198.6	204.0	206.2	1.43	<0.01	<0.01	0.28	-	-
84 d	218.3	224.3	225.8	1.86	0.02	<0.01	0.57	-	-
Body weight changes, kg ^2^	41.4	47.5	49.15	1.84	0.01	<0.01	0.50	-	-
Average daily gain, kg ^3^	0.493	0.570	0.585	0.021	<0.01	<0.01	0.60	<0.01	0.34
Supplement intake, g/d ^4^	63.7	62.1	60.7	1.74	0.33	0.18	0.50	<0.01	0.40

^1^ (1) mineral supplementation with no feed additives [**CONT**, n = 10]; (2) inclusion of 1400 mg of narasin/kg of supplement [**N1400**, n = 10]; or (3) inclusion of 2100 mg of narasin/kg of supplement [**N2100**, n = 10]. ^2^ Animals were weighed at the beginning of the experiment (d 0) and at the end of each period (d 28, 56, and 84) after a 16 h feed and water withdrawal, using the idBeck 3.0 electronic scale (Irmãos Beckhauser e Cia Ltd.a, Paranavaí, PR, Brazil). Body weight changes were calculated using the initial and final BW of each animal. ^3^ The average daily gain (kg/d) was calculated by dividing the gain obtained by the duration of each period (28 d). ^4^ The supplement was provided once a week, as well as the measurement of the refusal. The amount of supplement offered, and the refusal were quantified on a Toledo 9094C/4 electronic scale accurate to 1.0 g (Toledo do Brasil, São Bernardo do Campo, SP, Brazil). Supplements were offered in quantities sufficient to ensure at least 10% refusals, thereby permitting ad libitum intake. Samples of the supplement offered, and the leftovers were collected to determine the dry matter content (AOAC, 2000 [21]) for later calculation of the average supplement intake in the dry matter.

**Table 4:** Forage availability and chemical composition of the paddocks covered by *Urochloa brizantha* cv. Marandu in Exp. 2.

Item	Treatments ^1^		SEM	*p*-Value		
	CONT	N1400		TRT	Period	T × P
Forage availability, T DM/ha ^2^						
Initial	6.43	6.18	0.46	0.57	0.22	0.72
Final	5.44	5.60	0.47	0.68	0.10	0.84
Chemical composition ^3^						
Dry matter	20.56	21.17	0.44	0.28	0.29	0.26
Organic matter	89.11	89.21	0.12	0.44	<0.01	0.81
Crude protein	11.30	11.19	0.24	0.67	<0.01	0.96
Neutral detergent fiber	71.68	72.13	0.31	0.18	<0.01	0.53
Acid detergent fiber	35.99	36.35	0.27	0.30	<0.01	0.71

^1^ (1) mineral supplementation with no fed additives [**CONT**, n = 10] and (2) inclusion of 1400 mg of narasin/kg of supplement [**N1400**, n = 10]. ^2^ Total forage availability in the paddocks was assessed at the entry and exit of the animals, i.e., on d 1 and 28 of each period, respectively. The quantitative samples were harvested close to the ground using 0.25 m^2^ metallic frames (0.5 × 0.5 m) placed on the representative sites. ^3^ The samples obtained were sent to the laboratory for subsequent determination of the dry matter content and calculation of forage availability per hectare.

**Table 5:** Experiment 2: Performance and supplement intake of Nellore calves receiving a mineral supplementation with no feed additives (**CONT**) or inclusion of 1400 mg of narasin/kg of supplement (**N1400**).

Item	Treatments ^1^		SEM	*p*-Value		
	CONT	N1400		TRT	Period	T × P
*Body weight*, kg ^2^						
Initial	193.2	193.0	0.22	0.94	-	-
28 d	202.0	204.2	0.88	0.08	-	-
56 d	216.2	222.2	1.13	<0.01	-	-
84 d	234.8	243.0	1.32	<0.01	-	-
112 d	254.5	263.6	1.37	<0.01	-	-
Body weight changes, kg ^2^	61.34	70.61	1.35	<0.01	-	-
Average daily gain, kg ^3^	0.550	0.632	0.02	<0.01	<0.01	0.89
Supplement intake, g/d ^4^	64.07	64.94	1.76	0.25	<0.01	0.17

^1^ (1) Mineral supplementation with no feed additives [**CONT**, n = 10] and (2) inclusion of 1400 mg of narasin/kg of supplement [**N1400**, n = 10]. ^2^ The animals were weighed at the beginning of the experiment (d 0) and at the end of each period (d 28, 56, 84, and 112) after 16 h of feed and water withdrawal, using the idBeck 3.0 electronic scale (Irmãos Beckhauser e Cia Ltd.a, Paranavaí, PR, Brazil). Body weight changes were calculated using the initial and final BW of each animal. ^3^ The average daily gain (kg/d) was calculated by dividing the gain obtained by the duration of each period (28 d). ^4^ The supplement was provided once a week, as well as the measurement of the refusal. The amount of supplement offered, and the refusal were quantified on a Toledo 9094C/4 electronic scale accurate to 1.0 g (Toledo do Brasil, São Bernardo do Campo, SP, Brazil). Supplements were offered in quantities sufficient to ensure at least 10% refusals, thereby permitting ad libitum intake. Samples of the supplement offered, and the leftovers, were collected to determine the dry matter content (AOAC, 2000 [21]) for later calculation of the average supplement intake in the dry matter.

**Table 6:** Forage availability and chemical composition of the paddocks covered by *Urochloa brizantha* cv. Marandu in Exp. 3.

Item	Treatments ^1^		SEM	*p*-Value		
	PROT	PROT250		TRT	Period	T × P
Forage availability, T DM/ha ^2^						
Initial	5.64	5.71	0.29	0.82	<0.01	0.38
Final	4.70	4.71	0.29	0.97	<0.01	0.89
Chemical composition ^3^						
Dry matter	23.33	23.25	1.01	0.92	<0.01	0.94
Organic matter	88.54	88.44	0.20	0.58	<0.01	0.81
Crude protein	8.29	8.39	0.29	0.71	<0.01	0.91
Neutral detergent fiber	73.70	73.81	0.44	0.82	<0.01	0.99
Acid detergent fiber	37.21	37.38	0.33	0.72	0.03	0.46

^1^ (1) Protein supplementation with no feed additives [**PROT,** n = 15] and (2) inclusion of 250 mg of narasin/kg of protein supplement [**PROT250,** n = 15]. ^2^ Total forage availability in the paddocks was assessed at the entry and exit of the animals, i.e., on d 1 and 28 of each period, respectively. The quantitative samples were harvested close to the ground using 0.25 m^2^ metallic frames (0.5 × 0.5 m) placed on the representative sites. ^3^ The samples obtained were sent to the laboratory for subsequent determination of the dry matter content and calculation of forage availability per hectare.

**Table 7:** Experiment 3: Performance and supplement intake of Nellore yearlings receiving a protein supplementation with no feed additives (**PROT**) or inclusion of 250 mg of narasin/kg of protein supplement (**PROT250**).

Item	Treatments ^1^		SEM	*p*-Value		
	PROT	PROT250		TRT	Period	T × P
Body weight, kg ^2^						
Initial	332.0	331.8	0.13	0.58	-	-
28 d	339.2	340.7	0.77	0.29	-	-
56 d	346.0	348.1	0.76	0.08	-	-
84 d	358.4	361.5	0.80	0.02	-	-
112 d	357.7	363.0	0.84	<0.01	-	-
Body weight changes, kg ^2^	25.77	31.12	0.84	<0.01	-	-
Average daily gain, kg ^3^	0.230	0.278	0.016	0.04	<0.01	0.88
Supplement intake, g/d ^4^	435.3	433.3	12.13	0.74	<0.01	0.70

^1^ (1) Protein supplementation with no feed additives [PROT, n = 15] and (2) inclusion of 250 mg of narasin/kg of protein supplement [PROT250, n = 15]. ^2^ The animals were weighed at the beginning of the experiment (d 0) and at the end of each period (d 28, 56, 84, and 112) after a 16 h feed and water withdrawal, using the idBeck 3.0 electronic scale (Irmãos Beckhauser e Cia Ltda, Paranavaí, PR, Brazil). Body weight changes were calculated using the initial and final BW of each animal. ^3^ The average daily gain (kg/d) was calculated by dividing the gain obtained by the duration of each period (28 d). ^4^ The supplement was provided once a week, as well as the measurement of the refusal. The amount of supplement offered, and the refusal were quantified on a Toledo 9094C/4 electronic scale, accurate to 1.0 g (Toledo do Brasil, São Bernardo do Campo, SP, Brazil). Supplements were offered in quantities sufficient to ensure at least 10% refusals, thereby permitting ad libitum intake. Samples of the supplement offered, and the leftovers, were collected to determine the dry matter content (AOAC, 2000 [21]) for later calculation of the average supplement intake in the dry matter.

## Data Availability

The original contributions presented in this study are included in the article. Further inquiries can be directed to the corresponding author(s).

## Abbreviations

The following abbreviations are used in this manuscript:

ADG	Average daily gain
BW	Body weight
DMI	Dry matter intake
OM	Organic matter
CP	Crude protein
NDF	Neutral detergent fiber
ADF	Acid detergent fiber
